# Doctors as Leaders

**Published:** 1914-11

**Authors:** 


### Doctors as Leaders.

Everywhere the doctor is a leader, whether he be the medicine man of a savage tribe, or the humble family doctor, whose relations make him the repository of the inner life and the adviser in the daily relations of his clients. A community naturally looks to that man who ministers to them in time of need; the citizens naturally turn to him for direction, and no doctor fills the place that he should occupy unless he realizes this position, and then prepares to accept and fill it.

Having this opportunity for good, he is like the servant of the parable who buried his talent, unless he accepts and fills this responsibility.

Not only is it a duty for the doctor to fill this position of leadership; it is a potent factor in his success, should he so comport himself as to deserve his position. G. H. B.

